# Synthesis and Characterization of a Thermoresponsive Copolymer with an LCST–UCST-like Behavior and Exhibiting Crystallization

**DOI:** 10.1021/acsomega.3c03162

**Published:** 2023-08-17

**Authors:** Natalie Solfrid Gjerde, Alessandra Del Giudice, Kaizheng Zhu, Kenneth D. Knudsen, Luciano Galantini, Karin Schillén, Bo Nyström

**Affiliations:** † Department of Chemistry, “Sapienza” University of Rome, P.O. Box 34-Roma 62, Piazzale A. Moro 5, I-00185 Roma, Italy; ‡ Faculty of Engineering, Østfold University College, P.O. Box 700, 1757 Halden, Norway; § Institute for Energy Technology, P.O. Box 40, N-2027 Kjeller, Norway; ∥ Division of Physical Chemistry, Department of Chemistry, Lund University, P.O. Box 124, SE-221 00 Lund, Sweden; ⊥ Department of Chemistry, University of Oslo, P.O. Box 1033, Blindern, N-0315 Oslo, Norway

## Abstract

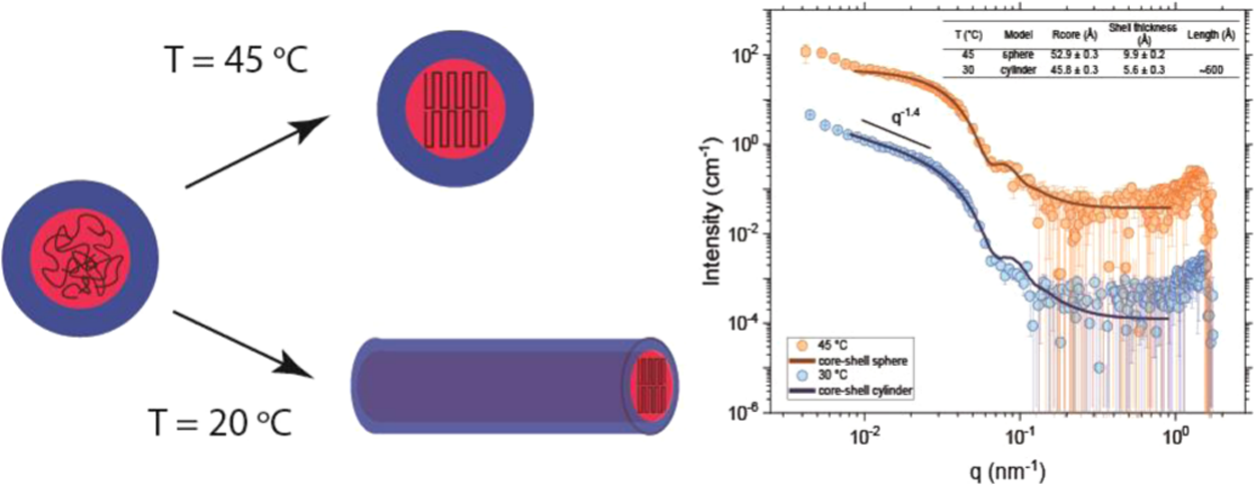

In this work, the diblock copolymer methoxy-poly(ethylene glycol)-*block*-poly(ε-caprolactone) (MPEG–*b*-PCL) was synthesized with a block composition that allows this polymer in aqueous media to possess both an upper critical solution temperature (UCST) and a lower critical solution temperature (LCST) over a limited temperature interval. The value of the UCST, associated with crystallization of the PCL-block, depended on heating (H) or cooling (C) of the sample and was found to be CP_UCST_^H^ = 32 °C and CP_UCST_^C^ = 23 °C, respectively. The LCST was not affected by the heating or cooling scans; assumed a value of 52 °C (CP_LCST_^H^ = CP_LCST_^C^). At intermediate temperatures (e.g., 45 °C), dynamic light scattering (DLS), small-angle X-ray scattering (SAXS), and cryogenic transmission electron microscopy (cryo-TEM) showed that the solution consisted of a large population of spherical core–shell particles and some self-assembled rodlike objects. At low temperatures (below 32 °C), differential scanning calorimetry (DSC) and wide-angle X-ray scattering (WAXS) in combination with SAXS disclosed the formation of crystals with a cylindrical core–shell structure. Cryo-TEM supported a thread-like appearance of the self-assembled polymer chains. At temperatures above 52 °C, incipient phase separation took place and large aggregation complexes of amorphous morphology were formed. This work provides insight into the intricate interplay between UCST and LCST and the type of structures formed at these conditions in aqueous solutions of MPEG–*b*-PCL diblock copolymers.

## 1 Introduction

It is well established that amphiphilic thermoresponsive copolymers can self-assemble into micelles when the concentration reaches above the critical micelle concentration (cmc) of the copolymer at a specific temperature or when the temperature exceeds the critical micelle temperature (cmt).^1,2^ The micelles that are formed may assume different shapes, such as spherical, cylindrical, wormlike, and vesicle-like.^3−13^ Amphiphilic copolymers have attracted substantial concern because of their potential use in the design of nanomaterials with organized structures and tunable features for various applications.^14,15^ Even the simple amphiphilic diblock copolymers, where both blocks are amorphous, have attracted excessive interest because of their ability to self-assemble into well-defined supramolecular architectures in solutions.^10,16,17^ A more complex self-assembly process emerges when one block of the diblock copolymer is able to crystallize.^10,18,19^ A typical copolymer in this category is methoxy-poly(ethylene glycol)-*block*-poly(ε-caprolactone), abbreviated as MPEG-*b*-PCL, where the PCL block has the ability to crystallize at a certain temperature.^20^ This copolymer has been employed in many drug delivery applications^21−25^ because of its biocompatibility and biodegradability. Most of these studies were focused on drug delivery features, and little attention has been devoted to temperature-induced phase transitions and morphological changes of the formed micelles and association complexes.

At moderate temperatures, the amorphous MPEG block is hydrophilic, and the PCL block is crystalline and hydrophobic with a polar ester group and five nonpolar methylene groups in its repeating unit. To obtain a water-soluble thermoresponsive copolymer that possesses both upper critical solution temperature (UCST) and lower critical solution temperature (LCST) behaviors in an easily accessible temperature range, it is important that the ε-caprolactone (CL)/ethylene glycol (EG) composition ratio of the copolymer is optimized.

In this work, we synthesized a copolymer with a CL/EG ratio that makes it possible to observe both UCST- and LCST-like behaviors over a convenient temperature interval. The aim of this study is to characterize the effect of temperature on the crystalline and amorphous regions of the copolymer by using turbidimetry, differential scanning calorimetry (DSC), and dynamic light scattering (DLS). Temperature-induced structural changes of crystallites and association complexes were established through small-angle X-ray scattering (SAXS) and wide-angle X-ray scattering (WAXS), as well as cryogenic transmission electron microscopy (cryo-TEM). From the results, a rather complex picture emerges about the behavior of the copolymer at different temperatures; this information should be important for the use of this copolymer for biomedical applications, such as carriers for drugs. To the best of our knowledge, this study is the first to describe in structural detail the temperature-dependent reorganization and morphological changes of an MPEG–*b*-PCL system possessing both upper and lower critical solution temperatures.

## 2 Experimental Section

### 2.1 Materials

ε-Caprolactone from Sigma-Aldrich was dried and distilled over CaH_2_ under reduced pressure and stored in a refrigerator. Methoxy-poly(ethylene glycol) monoether (Sigma-Aldrich) has a number-average molecular weight of 750 g/mol. Stannous 2-ethylhexanoate (stannous octoate, Sn(Oct)_2_) was purchased from Sigma-Aldrich and used as received without further purification. All other chemicals were reagent-grade and used as obtained.

### 2.2 Polymer Synthesis

**Figure 1:**
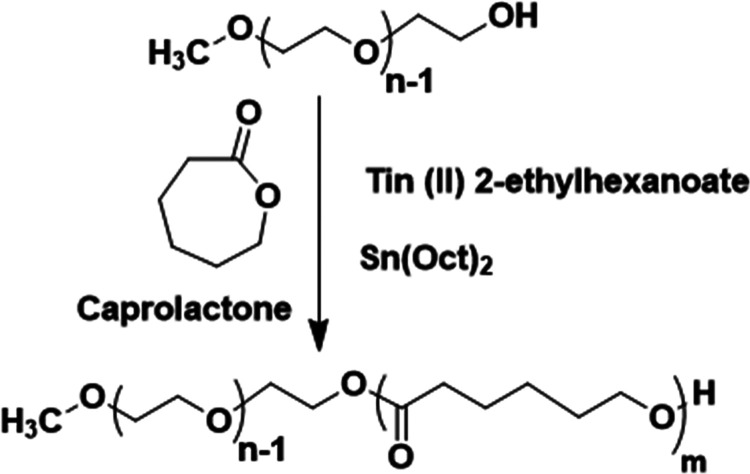
Synthetic route for the preparation of the diblock copolymer MPEG-*b*-PCL via a ring-opening polymerization procedure.

The diblock copolymer, MPEG-*b*-PCL, was prepared by ring-opening polymerization (ROP) of ε-caprolactone with MPEG as the initiator and stannous octoate as the catalyst.^25−29^ The synthetic procedure is outlined in Figure 1.

The synthesis was conducted in the following way: MPEG (25 g, 33.3 mmol) was dissolved in anhydrous toluene (80 mL), and the solvent was azeotropically distilled off to a final volume of 20 mL to remove the residual water adsorbed by the polymer. When the temperature of the flask had decreased to room temperature, ε-caprolactone (33 g, 0.29 mol) and stannous octoate (0.15 mmol, 0.15 g in dried toluene (0.5 mL)) were added to the reaction mixtures and stirred at 120 °C for 24 h in an atmosphere of argon for protection. The flask was then cooled to room temperature; the reaction mixture was dissolved in dichloromethane (50 mL), and the polymer was allowed to precipitate into cold diethyl ether and the procedure was repeated three times. The white solid product (55 g) was finally obtained with a yield of 95% after drying in vacuum at RT for another 48 h.

**Figure 2:**
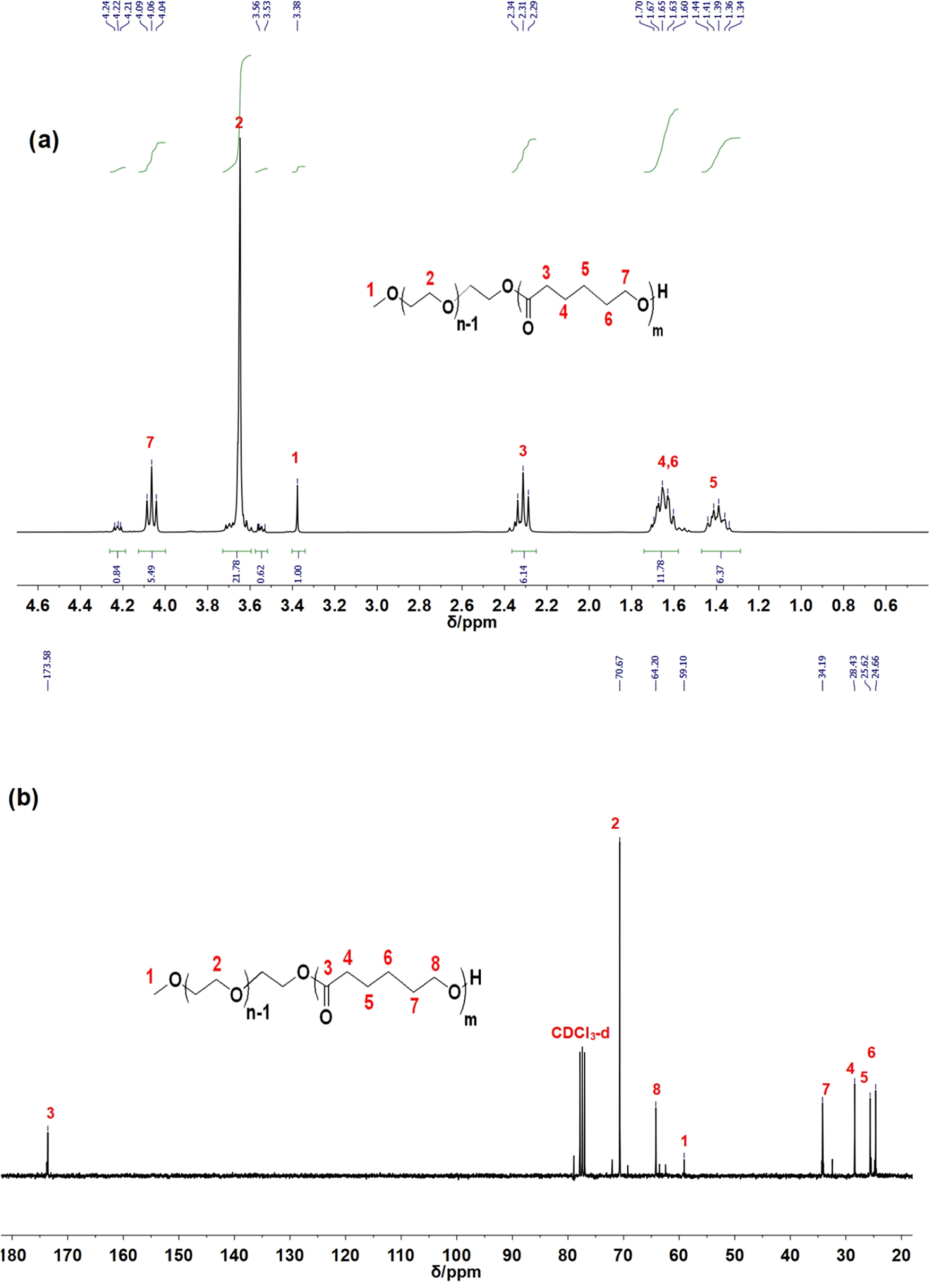
NMR characterization of MPEG-*b*-PCL diblock copolymer by using (a) ^1^H NMR spectrum (CDCl_3_-*d* as a solvent, 400 MHz) and (b) ^13^C NMR spectrum (CDCl_3_-*d* as a solvent, 150 MHz).

The chemical structure and composition of the diblock copolymer were determined by using ^1^H NMR and ^13^C NMR measurements (2056 scans) on CDCl_3_ solutions containing tetramethylsilane (TMS) as the reference at 25 °C (Bruker AVANCE II 400 MHz spectrometer), and the results are displayed in Figure 2. Both the proton and ^13^C NMR spectra show that the sample is pure, without any impurity/unwanted peaks (residue solvent, etc.). The molar composition of the sample was calculated by comparing the integral area of the capped methoxyl signal (**1**) of MPEG (δ = 3.38 ppm), the methylene group of PEG (**2**), and the CL methylene group (**3**) (δ = 2.30 ppm) (Figure 2a). The peak at δ = 174 ppm indicates the incorporation of carboxyl groups (**3**, **C=O**) into the polymer backbone (Figure 2b). To indicate the composition of ε-caprolactone and ethylene glycol in the repeating units of the synthesized diblock copolymer, the chemical formula can be written as MP(EG)*_m_*-*b*-P(CL)*_n_*, where the indices m and n were estimated to be 17 and 9, respectively. Therefore, the diblock copolymer can be abbreviated as MP(EG)_17_-*b*-P(CL)_9_.

GPC was performed on a Tosoh Eco-SEC dual detection (RI and UV (195–350 nm)) GPC system coupled to an external Wyatt Technologies miniDAWN Treos multiangle light scatting (MALS) detector. Samples dissolved in tetrahydrofuran (THF) were run at a flow rate of 0.5 mL/min at 35 °C. The MZ-Gel SD plus linear column was used in this work. The refractive index increment value (d*n*/d*c* = 0.0610 mL g^–1^) was determined from online measurements assuming 100% mass recovery (RI was used as a concentration detector) using the Astra 6 software package (Wyatt Technologies) by selecting the entire trace from the analyzed peak to the onset of the solvent peak or flow marker.^30^ Absolute molecular weights and molecular weight distributions were calculated using the Astra software package. The concentration of the polymer samples is 10.0 mg/mL. Figure S1 (Supporting Information) shows the GPC curve of the copolymer, and the results reveal a low polydispersity (*M*_w_/*M*_n_ = 1.17) and a weight-average molecular weight of *M*_w_ = 2600 g/mol. The elution peak is symmetric, and it exhibits no tails on the low-molecular-weight side.

### 2.3 Turbidity

The turbidity measurements on the diblock copolymer sample were carried out by employing an NK60-CPA cloud point analyzer from Phase Technology. The phase change taking place when the temperature was varied (both heating and cooling scans were carried out) was probed by a scanning diffusive light scattering technique with high sensitivity. A beam from the light source AlGaAs (654 nm) from Phase Technology was focused on the considered sample. The optical system above the sample monitors the scattered intensity signal (*S*) at various temperatures, and the relation between the calculated turbidity (τ) and *S* from the cloud point analyzer was given by^31^ τ(cm^–1^) = 9.0 × 10^–9^*S*^3.751^. The sample was applied onto a specially designed plate that is coated with a thin metallic layer that is working as a high reflectivity mirror. To control and change the temperature quickly and accurately, the instrument is equipped with a thermoelectric device consisting of an array of Peltier elements. The experiments were performed over an extended temperature interval (15–60 °C).

### 2.4 Differential Scanning Calorimetry (DSC)

The crystallization process of 1 wt % aqueous solutions of MP(EG)_17_-*b*-P(CL)_9_ was studied by DSC. The measurements were conducted by using a Mettler Toledo, Switzerland, DSC822e fitted with a MultiSTAR HSS7 sensor. The sample was preheated to 60 °C and allowed to equilibrate for 1 h before proceeding with the measurement to ensure the removal of all crystals and a clean state of the sample history. Then, the cooling curve from 60 °C down to 15 °C was measured at a rate of 0.2 °C/min. The sample was then allowed to stay at 15 °C for 1 h before measuring the heating curve from 15 to 60 °C with the same measuring rate of 0.2 °C/min. The experiment was repeated on 3 samples with the same polymer concentration; overlapping heating/cooling curves were obtained. The measurements were carried out on degassing samples.

### 2.5 Dynamic Light Scattering

DLS measurements were carried out on a Malvern Nano-Zeta-Sizer instrument equipped with a 5 mW HeNe laser (λ = 632.8 nm) and a digital logarithmic correlator. The normalized intensity autocorrelation functions were measured at a scattering angle of 173°. The temperature of the copolymer solutions was accurately controlled by using a Peltier device in the instrument (±0.1 °C). The correlation functions were analyzed with the aid of a non-negative least-squares fitting approach to obtain the intensity-weighted distributions of the apparent hydrodynamic radii (*R*_H_).

### 2.6 Small- and Wide-Angle X-ray Scattering

Small- (SAXS) and wide-angle (WAXS) measurements were carried out at SAXSLab Sapienza with a Xeuss 2.0 Q-Xoom system (Xenocs SA, Grenoble, France) equipped with a microfocus Genix 3D X-ray source (wavelength λ = 1.542 Å) and a two-dimensional Pilatus 3R 300 K detector (Dectris Ltd., Baden, Switzerland), which can be placed at a variable distance from the sample and with an additional Pilatus 3 R 100 K detector at a fixed shorter distance from the sample. The wave vector *q* = (4π/λ)sin(θ/2), where θ is the angle between the scattered beam and the incoming beam, was calibrated using silver behenate. By using the two-pinhole collimation system equipped with “scatterless” slits, the beam size was set to 0.5 × 0.5 mm. Samples were loaded into vacuum-tight quartz capillary cells with a thickness of 1.5 mm, and the measurements were conducted in the instrument sample chamber under reduced pressure (∼0.2 mbar) at temperatures in the range of 20–45 °C. The two-dimensional scattering patterns were subtracted for the “dark” counts, masked, azimuthally averaged, and normalized for the transmitted beam intensity, exposure time, and subtended solid angle per pixel by using the FoxTrot software developed at SOLEIL. The profiles of the scattered intensity versus *q* were subtracted for the contributions from water and cell, and the intensity was put on an absolute scale (cm^–1^) by dividing for the known thickness. The different angular regions were merged with the aid of the SAXS-utilities tool.^32^ The peaks in the scattered intensity curves from WAXS were interpreted as Bragg peaks of the crystalline structures and reflections generated by the coassembled structures. The sample was observed equilibrated at 45 °C and kept for 6 h while both SAXS and WAXS measurements were carried out simultaneously. The sample was then cooled to 30 °C and observed for 6 h using both techniques.

### 2.7 Cryogenic Transmission Electron Microscopy

Cryo-TEM measurements were carried out on a JEM-2200F transmission microscope (JEOL) at the National Center for High Resolution Electron Microscopy (nCHREM) at Lund University, Sweden. This instrument is a JEM-2200FS transmission electron microscope (JEOL) that is specially designed for cryo-TEM with low-dose imaging and tomography. The instrument is armed with a field-emission electron source, a cryo pole piece in the objective lens, and an omega filter to ensure energy-filtered transmission electron microscopy. Zero-energy loss images were taken up at an acceleration voltage of 200 kV by using a bottom-mounted TVIPS F416 camera under low-dose conditions. For the preparation of specimens, a Leica EM GP automatic plunge freezer system (from Leica Microsystems, Stockholm, Sweden) was employed with the environmental chamber set to 20 or 45 °C. A 4 μL droplet of the sample solution (1 wt %) was deposited onto a lacey Formvar carbon-coated grid and was blotted for 2 s with filter paper to remove excess liquid. At the 45 °C condition, 2 μL of the preheated sample solution was instead deposited due to the increased solution viscosity. The grid was thereafter plunged into liquid ethane (a temperature of about 183 °C) to make the vitrification fast and to keep the sample in its native state. Then, the specimens were stored in liquid nitrogen (−196 °C) and subsequently transferred to the microscope.

## 3 Results and Discussion

### 3.1 Turbidity

Turbidimetry is a powerful method to monitor the formation of temperature-induced association complexes and reveal systems approaching macroscopic phase separation. Thermoresponsive polymers belong to a class of materials that can undergo reversible alteration in their solubility at a particular temperature called the critical transition temperature. This feature is usually utilized to classify thermosensitive polymers into one of two classes depending on whether they phase-separate from the solvent as the temperature increases, displaying an LCST, or that the polymers turn out to be solvent-miscible with increasing temperature.^33−35^ In the latter case, the system shows an upper critical solution temperature (UCST). Most of the studies reported so far have focused on thermoresponsive polymers exhibiting an LCST type of behavior. One of the most studied LCST types of polymers is poly(*N*-isopropylacrylamide) (PNIPAAM) in aqueous media; in addition to this gold standard with an LCST at approximately 32 °C,^36^ there are reports on a number of poly(ethylene glycol)-functionalized methacrylate polymers, including a triblock copolymer of PEO and poly(propylene oxide), that are reported to show an LCST behavior.^37−39^ In this work, a MPEG–*b*-PCL copolymer was synthesized with a composition (MP(EG)_17_-*b*-P(CL)_9_) that allows the polymer to exhibit both an LCST and a UCST behavior over a limited temperature interval.

**Figure 3:**
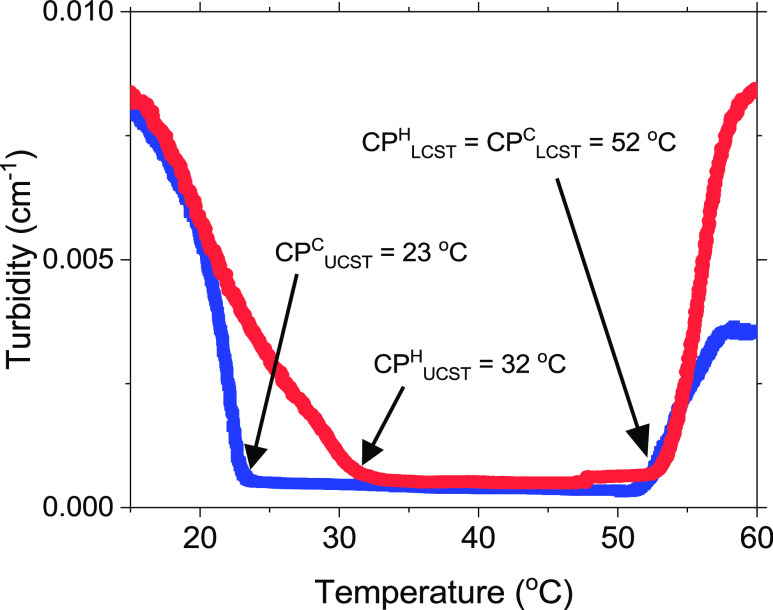
Turbidity curves of 1 wt % of MP(EG)_17_-*b*-P(CL)_9_ in water. The blue curve represents the cooling curve of the sample, and the red curve represents the heating curve. In both series, a heating/cooling rate of 0.2 °C/min was used.

Figure 3 illustrates the turbidity during both heating and cooling of a 1 wt % aqueous solution of MP(EG)_17_-*b*-P(CL)_9_. Let us first discuss the heating curve (red curve), where the sample is heated from 15 °C up to 60 °C. The high turbidity observed at low temperatures indicates the formation of large aggregates, which eventually will lead to a macroscopic phase separation. It is known that PCL in the MPEG–*b*-PCL block copolymer can crystallize^10,40,41^ in aqueous solution and form semicrystalline micelles, with a crystalline core of PCL and an MPEG corona. At low temperatures, the situation is controlled by the crystallization-driven self-assembly and formation of aggregates in the MPEG–*b*-PCL system and strong packing interactions between PCL blocks. As the temperature increases from 15 to 30 °C, the crystals are gradually broken up and disrupted. The transition temperature for the cloud point CP_UCST_^H^ upon heating was found to be 32 °C. In the temperature range from ca. 32 to above 50 °C, the copolymer is completely solvent-miscible, and the solution is fully transparent. The transition temperature for the cloud point CP_LCST_^H^ was observed to be 52 °C. Above this temperature, the turbidity increases strongly, suggesting the formation of large aggregates and ultimately macroscopic phase separation takes place. Because of this fast phase separation with the precipitation of polymer, the rest of the measurements have been carried out at temperatures up to 52 °C because scattering techniques are not applicable at higher temperatures. In an earlier study^26^ on MPEG–*b*-PCL with a similar composition as in this work, a phase diagram with two phases was observed at temperatures above ca. 50 °C and at temperatures below ca. 30 °C. These findings are consistent with our results for CP_UCST_^H^ and CP_LCST_^H^.

When the polymer solution undergoes cooling (blue curve in Figure 3) a pronounced hysteresis effect is observed at low temperatures. In this case, a much lower cloud point CP_UCST_^C^ of 23 °C is detected. It seems that a lower temperature is needed before the crystals start to grow. This indicates that the melting process of the crystals needs a higher temperature than forming the crystals, but we should also bear in mind that the kinetics of forming and melting the crystals may operate on different time scales. When it comes to the LCST, the heating and cooling cycles virtually yield the same values (ca. 52 °C) of CP_LCST_^H^ and CP_LCST_^C^. This indicates that the formation and disruption of the amorphous aggregates are less complex than the crystallization process at low temperatures.

### 3.2 Thermal and Crystallization Behaviors

**Figure 4:**
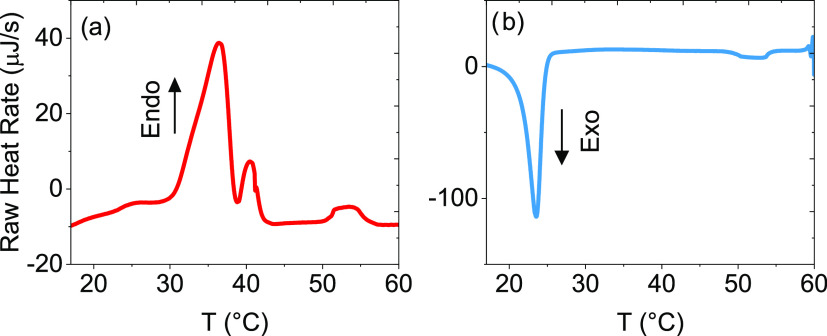
DSC heating (a) and cooling (b) curves of 1 wt % of MP(EG)_17_-*b*-P(CL)_9_ in water. The heating and cooling rates were set at 0.2 °C/min.

The crystallization and melting behaviors of MP(EG)_17_-*b*-P(CL)_9_ were investigated with the aid of DSC, and the results are displayed in Figure 4. The cooling of the 1 wt % MP(EG)_17_-*b*-P(CL)_9_ solution reveals an exothermic peak that is located at about 23 °C; this is a characteristic peak of crystallization. It is interesting to note that this temperature is the same as that reported for CP_UCST_^C^. This suggests that the upturn at low temperature of the blue turbidity curve in Figure 3 signals the formation of crystals. In a previous study^40^ on MPEG–*b*-PCL of various compositions, values of the crystallization temperature in the region of 27.6–32.2 °C were reported. The general trend at a constant MPEG block length was that the temperature increased with an increasing PCL block length. A similar observation was reported by X-ray diffraction (XRD) on MPEG–*b*-PCL diblock copolymers, i.e., the total degree of crystallinity of the copolymer increased from 7 to 23% as the length of the PCL block increased.^41^ This tendency suggests that most of the crystallinity of the block copolymer is governed by the PCL segments.

In the heating process, a pronounced endothermic peak is located at approximately 35 °C; this melting temperature is close to the value (32 °C) of CP_UCST_^H^ observed in the turbidity measurements discussed above. This suggests that the PCL domains melted at temperatures higher than CP_UCST_^H^. In a previous study^41^ on solutions (20 wt %) of MPEG–PCL of different compositions, the melting temperature increased from 45 to 50 °C as the PCL block length increased. The reason for the much lower melting temperature (32 °C) observed in this work may be due to the much shorter length of the PCL block and the lower (1 wt %) polymer concentration in the present study. There is also a weaker secondary endothermic peak located at 39 °C; a secondary peak has been argued^40^ to evolve when the length of the two blocks is significantly different as in this work; it was suggested to be related to the melting of the crystal phases of MPEG segments.^41^ Moreover, the small broad endothermic peak visible at approximately 52 °C (where turbidity measurements disclosed CP_LCST_^H^ = CP_LCST_^C^ = 52 °C), both upon heating and cooling, may be associated with the poorer thermodynamic conditions for MPEG at elevated temperatures leading to some ordered packing of the MPEG blocks at the PCL core and structural changes of the micelles prior to the macroscopic liquid–liquid phase separation.^39^ Most of the MPEG-generated association complexes observed by turbidity at high temperatures can be ascribed to the formation of mainly amorphous material.

### 3.3 Dynamic Light Scattering and Kinetics

**Figure 5:**
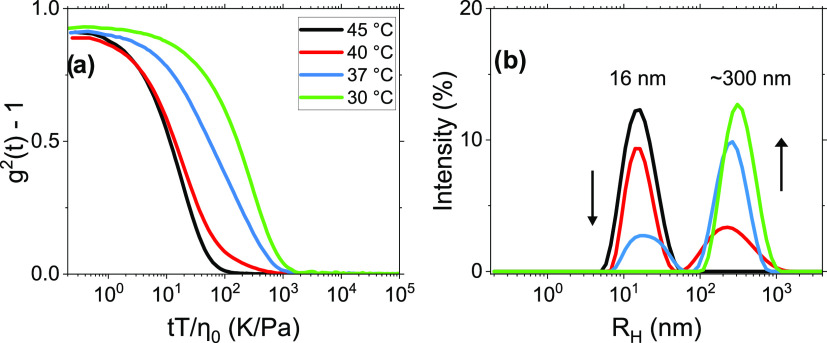
(a) Autocorrelation functions of the scattered intensity for 1 wt % of MP(EG)_17_-*b*-P(CL)_9_ aqueous solution at the temperatures indicated. (b) Intensity-weighted distributions of the apparent hydrodynamic radii at different temperatures.

The formation of crystals is a kinetic process that can be monitored by employing DLS. Figure 5a shows normalized autocorrelation functions of the scattered intensity for a 1 wt % of MP(EG)_17_-*b*-P(CL)_9_ aqueous solution at various temperatures. It is evident that the decay of the correlation function is shifted toward longer times as the crystallization-induced clusters grow with a decreasing temperature. At 45 °C, the relaxation is monomodal (Figure 5b), suggesting micelles with a hydrodynamic radius of ca. 16 nm. This size is close to that reported for other MPEG–*b*-PCL micelles.^20^ At 40 °C, bimodal relaxation modes are observed, where the fast mode represents micelles and the slow mode characterizes clusters of crystals with an average hydrodynamic radius of approximately 300 nm. At 37 °C, there are still two relaxation modes giving virtually the same sizes as at 40 °C, but in this case, the large crystal clusters are dominating. At 30 °C, the fast mode fades away; the relaxation process is controlled by the slow mode, and very large clusters of crystals dominate the behavior. This situation arises because the number and size of the formed aggregates grow and consequently the number of the small entities (micelles) strongly declines to the extent that it is not possible to detect the fast mode. This can be rationalized in terms of Ostwald ripening,^42^ or coarsening, as a basis to describe the cluster or crystal growth process. The phenomenon can be detected in, e.g., liquid sols, representing the alteration of an inhomogeneous structure over time, i.e., small crystals redeposit onto larger crystals.^43−45^ In crystallization, ripening is the process in which large crystals grow at the expense of smaller ones that shrink and ultimately disappear. The driving force in the coarsening process is the fact that larger particles are more energetically favored than smaller particles, leading to an apparent higher solubility for the smaller ones. The trend observed in Figure 5b can easily be rationalized in the framework of Ostwald ripening.

**Figure 6:**
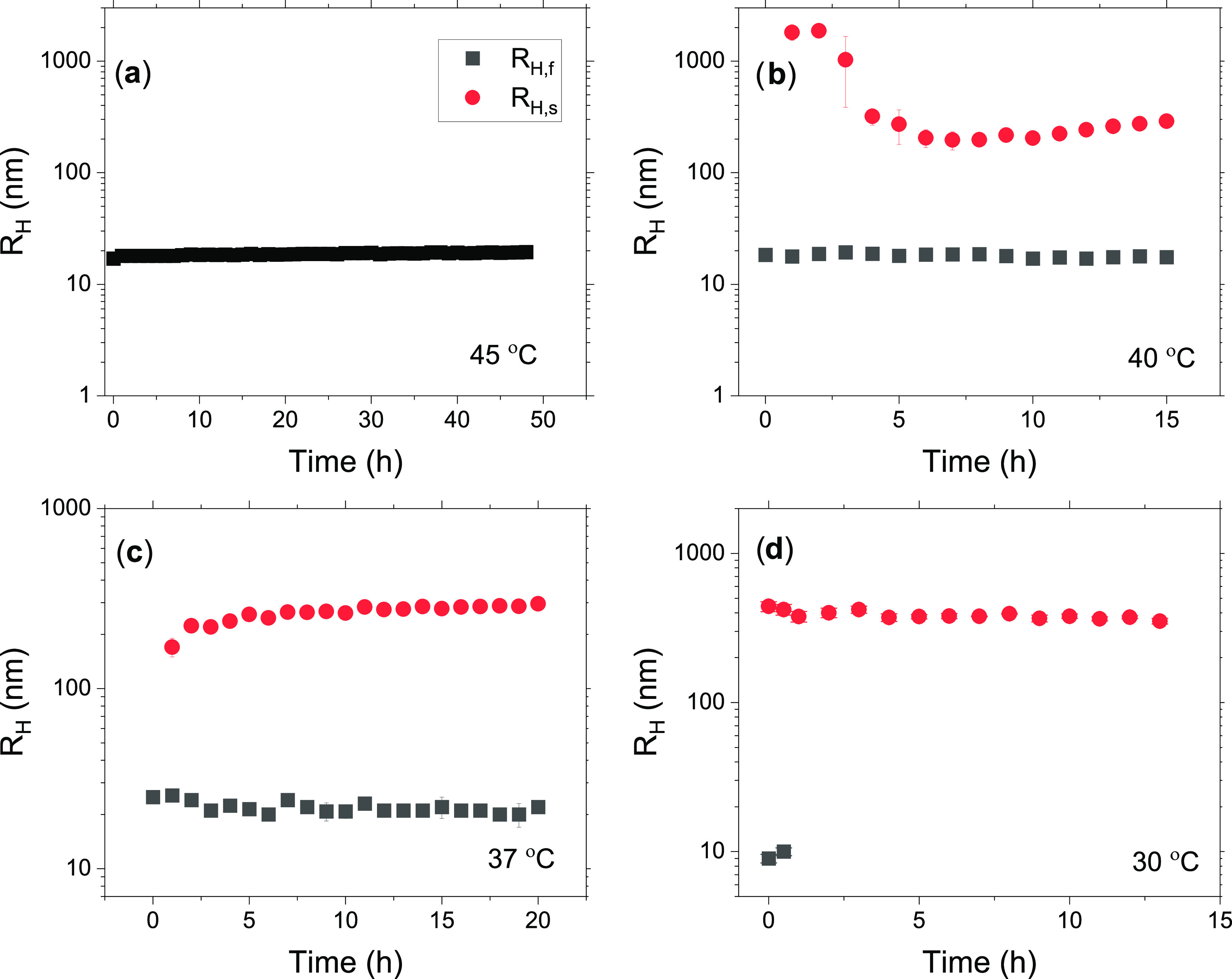
Time evolution of the fast and slow hhydrodynamic radii corresponding to the fast (*R*_H,f_) (black) and the slow mode (*R*_H,s_) (red) in 1 wt % of aqueous solutions of MP(EG)_17_-*b*-P(CL)_9_ at the temperatures indicated: (a) 45 °C, (b) 40 °C, (c) 37 °C, and (d) 30 °C.

Another issue that is interesting to elucidate is the growth of the crystals during time evolution. Figure 6 shows the effect of time on the particle size at different temperatures. At 45 °C, only the fast relaxation mode is discernible. At this temperature, micelles are the only species visible in solution. Their apparent hydrodynamic radius is stable over a long time. Already at 40 °C, the fast and slow relaxation modes are both visible, where the fast mode probes the micelles, and the slow mode can be attributed to large clusters during the formation of crystals. During the first 5–7 h, a contraction of the clusters is evident; this may suggest that ordering of the structure occurs initially in the process of forming crystals. At 37 °C, there are still two relaxation modes representing micelles and crystal forming structures, but in this case, the crystals are slowly growing over a long time, whereas the size of the micelles is practically constant. At 30 °C, the micelles disappear after a short time because of Ostwald ripening, and gigantic crystals are evolved. It is interesting to note that the size of the crystals is virtually constant over a long time; this indicates that the overall structure of the crystals is also intact over time. Distributions of the hydrodynamic radii at different temperatures, which are informative of the relative contribution to the scattered intensity of the small (micelles) and large (crystals) populations, are illustrated in Figure S2 (Supporting Information).

**Figure 7:**
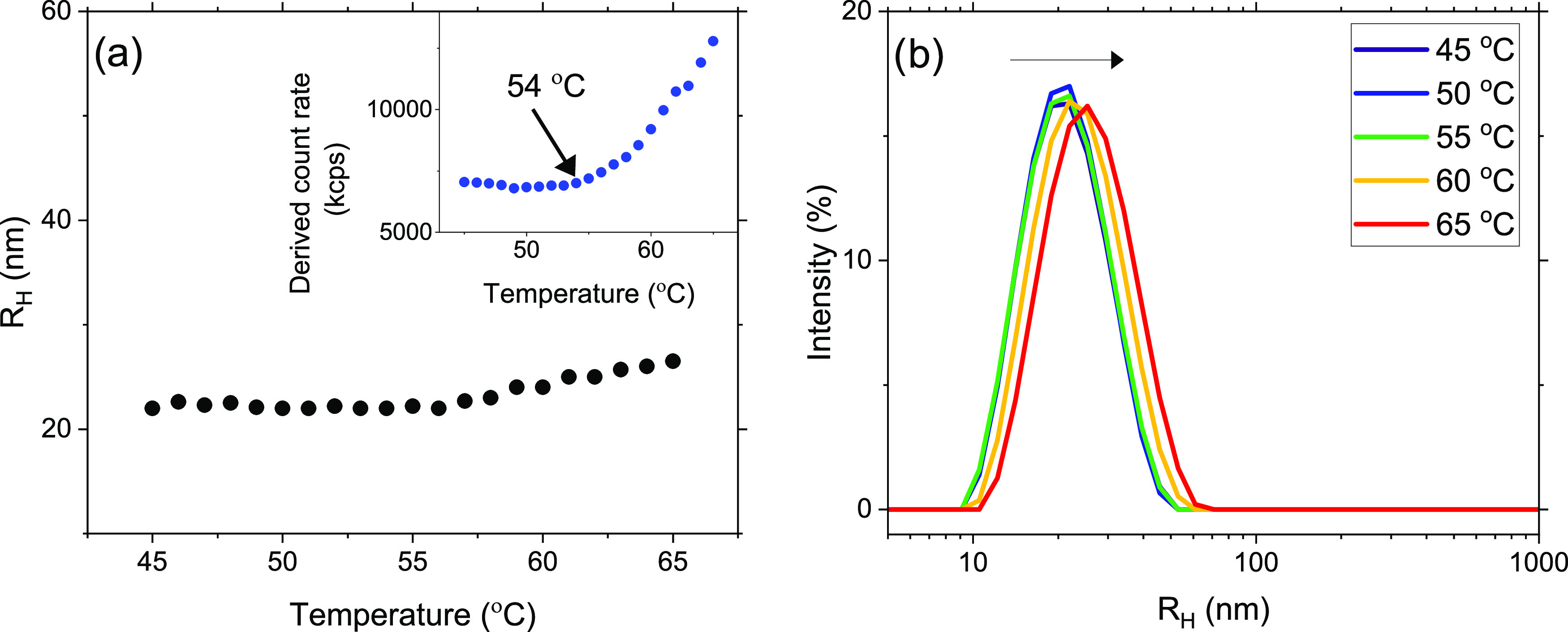
(a) Evolution of the apparent hydrodynamic radius (*R*_H_) upon heating 1 wt % of aqueous solutions of MP(EG)_17_-*b*-P(CL)_9_ from 45 to 65 °C. The inset plot shows significant increase of the count rate from 54 °C and above. (b) Intensity-weighted size distributions at the selected temperatures showing the presence of only one population in the sample. A slight increase in *R*_H_ can be observed at higher temperatures.

It has been established from the turbidity measurements that this MPEG–*b-*PCL block copolymer/water system has an LCST at high temperatures and that association complexes are formed with a more amorphous structure, according to DSC. Figure 7 depicts the temperature dependence of *R*_H_ in the high-temperature range where aggregates are formed. At approximately 54 °C, *R*_H_ increases with increasing temperature, indicating the formation of aggregates. This is further supported by the increase of the count rate above 54 °C (the inset figure). It is interesting to note that the aggregates in this case are much smaller than those formed during the formation of crystals as discussed above.

### 3.4 Local Structures and Crystallinity by SAXS and WAXS

**Figure 8:**
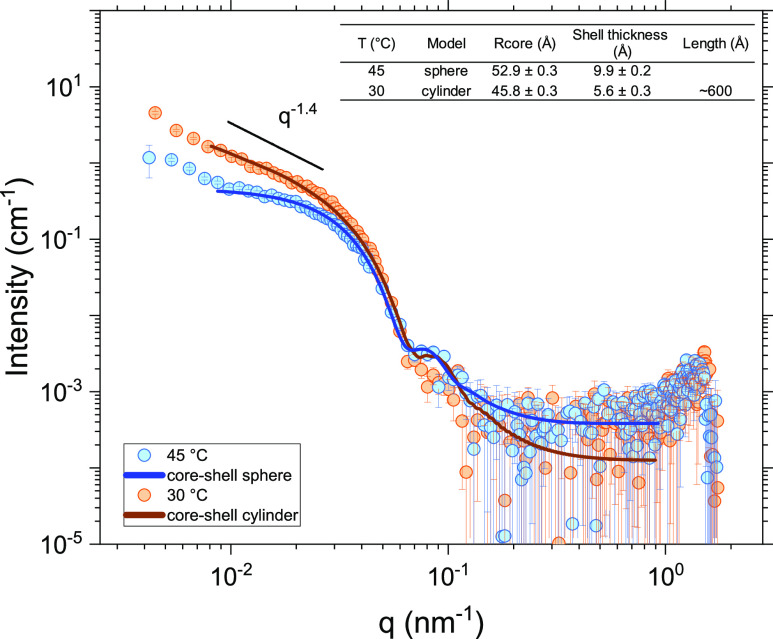
SAXS curves (every 4th point is skipped) for 1 wt % of MPEG–*b*-PCL aqueous solution at 30 and 45 °C.

In this work, we have employed SAXS to probe the local structures of the entities in 1 wt % aqueous solutions of MP(EG)_17_-*b*-P(CL)_9_ at different temperatures. The high-temperature SAXS data (at 45 °C) can be fitted effectively in the framework of a spherical core–shell model if the lowest *q*-range is disregarded (below about 0.09 nm^–1^). The core diameter is determined to be 10.6 ± 0.1 nm, and the shell thickness is ca. 1 nm (see Figure 8); thus, the overall size is approximately 12.6 ± 0.1 nm. This matches well with the cryo-TEM data (the average size of the particles in the cryo-TEM image at 45 °C is 15 nm) at the same temperature (Figure 10). However, it should be noted that the SAXS intensity shows an upturn at low *q*-values. This indicates that we have larger moieties falling outside the SAXS window. The hypothesis is that the MPEG chains form the corona and the PCL segments constitute the core in the large population of spherical core–shell particles. However, a close inspection of the cryo-TEM image reveals a minor population of self-assembled rodlike entities. This may be a preliminary stage of crystallization, occurring at lower temperatures. This process may explain why the value of the hydrodynamic diameter (ca. 32 nm) determined from DLS is significantly larger compared to the geometrical diameter of the spherical model. DLS provides a different-sized average (*Z*-average) that also includes the thickness of the solvation layer of the particle.

At 30 °C, the slope changes considerably; the data cannot be fitted with a spherical model even if polydispersity effects are taken into account. In this case, the SAXS data can be fitted with the aid of a model representing a core–shell cylinder object (log–log slope close to −1.4 in the scattering curve is typical for extended structures). Fitting of the scattering data with this model yields a core radius of 4.6 nm and a shell thickness of 0.6 nm. The length of the cylinder is estimated to be approximately 60 nm, but this value is uncertain due to the limited *q*-range available. The details of the spherical core–shell and core–shell cylinder models have been described elsewhere;^46^ the parameters considered in the modeling are summarized in the Supporting Information. The observation of an extended cylinder-like object is in accordance with the picture emerging from the cryo-TEM image at 20 °C (see the discussion below).

**Figure 9:**
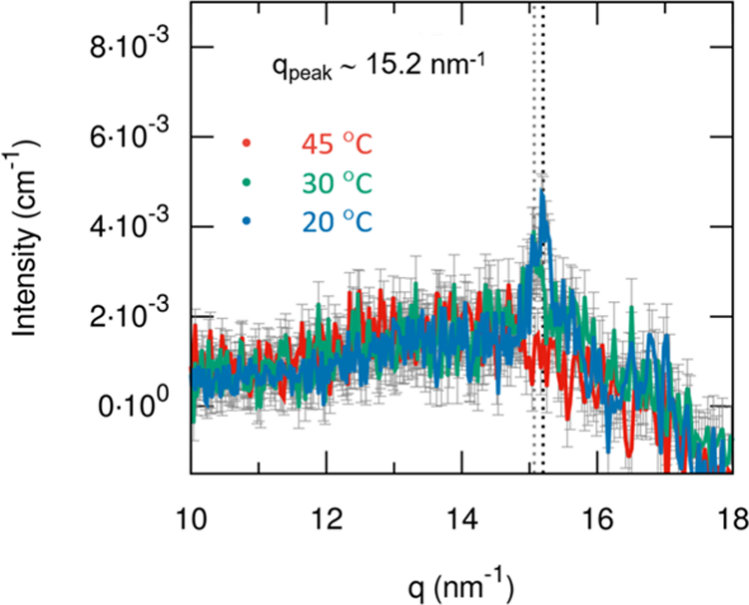
WAXS scattering curves of 1 wt % of MPEG–*b*-PCL aqueous solution at 45 °C (red), 30 °C (green), and 20 °C (blue).

To explore the short-range crystalline structure of the diblock copolymer in aqueous media, WAXS experiments were carried out. The results from the WAXS measurements are depicted in Figure 9. Although the signal is weak due to the relatively small amount of crystalline material compared to the surrounding liquid, a characteristic peak is observed at 15.2 nm^–1^, which corresponds to a repeating distance of 2π/(15.2 nm^–1^) = 0.41 nm or 4.1 Å. Such a distance may reflect the average lateral distance between stacked PCL chains in the core;^47^ this distance may signalize a crystal plane diffraction of PCL and thereby further confirm the occurrence of crystalline behavior at temperatures below UCST. The amplitude of the peak increases over a long time and a second (weaker) peak emerges located at 16.8 nm^–1^. Both peaks are more pronounced at the lowest temperature (20 °C) (Figure S3, Supporting Information). In a recent study^48^ on the bulk homopolymer PCL, the two characteristic peaks (15.2 and 16.8 nm^–1^) were observed and in addition a much weaker peak at 15.6 nm^–1^ that is not detectable in this work. It has been reported^49^ that bulk PCL takes a planar zigzag chain conformation in the crystalline phase.

### 3.5 Morphological Characterization with Cryo-TEM

**Figure 10:**
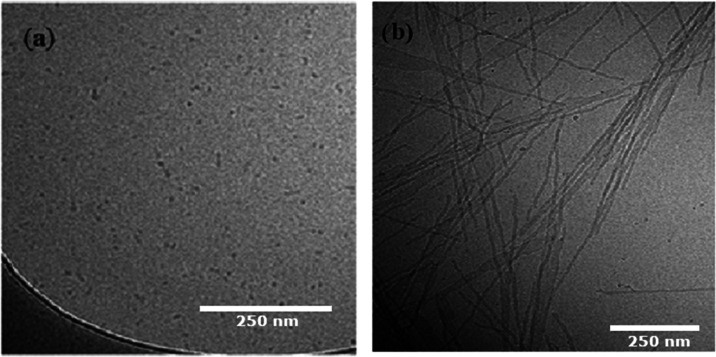
Cryo-TEM images of 1 wt % of MPEG–*b*-PCL aqueous solution at (a) 45 °C and (b) 20 °C.

Figure 10 shows cryo-TEM images at two different temperatures. At 45 °C, a large population of small sphere-like particles appear, but there seem to also be present a few rodlike objects of self-assembled micelles (Figure 10a). This may be responsible for the upturn of the scattered SAXS intensity at low *q*-values and the larger size observed from DLS.

At 20 °C (Figures 10b and S4 (Supporting Information)), long cylinders were formed as predicted from the SAXS results at 30 °C. It is obvious that the morphology is completely different from that at 45 °C, where mainly spherical core–shell moieties were found. A few lamellar-like structures were also detected (see Figure S4 (Supporting Information)). A similar feature was reported for the PEO-*b*-PCL diblock copolymer.^50^ Such a hierarchical self-assembly of PEO-*b*-PCL micelles in water is enhanced upon the incorporation of added PCL homopolymer.^51^ The long thread-like chains thus contribute to the formation of crystals as was observed in the DSC and WAXS findings. In a recent study^12^ on a PEO–PCL diblock copolymer, cylindrical structures were found to form at low temperatures (20 °C), and it was argued that the cylinders had a segmented or helical structure. The cryo-TEM image presented in Figure 10b shows long wiggled structures that support this hypothesis.

In this study, we have focused our attention on the temperature-induced self-assembling of the diblock copolymer MPEG-*b*-PCL in dilute solutions (1 wt %), but at higher polymer concentrations, it is possible to observe a temperature-induced sol–gel–sol transition and it was reported that the gel exhibited an interesting morphology.^41^ Another exciting behavior of this type of system that has attracted attention is the interaction mechanism and ionic conductivity of electrolytes composed of lithium perchlorate (LiClO_4_) and MPEG-*b*-PCL diblock copolymer. At certain compositions of the LiClO_4_/MPEG-*b*-PCL polymer electrolyte, DSC analyses revealed the occurrence of a macroscopic phase separation.^52^ An interesting study^53^ of thermoresponsive diblock copolymers described copolymers consisting of a nonionic block, which displays LCST behavior and a zwitterionic block that exhibits UCST. The transition temperature for the UCST behavior can be modulated by the salt concentration in the solution.

## 4 Conclusions

In summary, we synthesized the diblock copolymer MP(EG)_17_-*b*-P(CL)_9_ with a block composition that yielded both an upper critical solution temperature and a lower critical solution temperature over a limited temperature region. The cloud points were found to be CP_UCST_^C^ = 23 °C and CP_UCST_^H^ = 32 °C and CP_LCST_^H^ = CP_LCST_^C^ = 52 °C. At intermediate temperatures (e.g., 45 °C), SAXS indicates that we have small spherical core–shell entities and some larger complexes; together with DLS and cryo-TEM, the conjecture is that a large population of these small moieties coexist with a minor fraction of short rods.

At low temperatures (below 32 °C), crystals are formed, and this is supported by DSC and WAXS measurements. SAXS experiments demonstrate that the copolymer at low temperatures forms core–shell cylinder particles. Cryo-TEM reveals the existence of long thread-like chains. At temperatures above CP_LCST_, an incipient phase separation takes place, and aggregates are formed with a more amorphous morphology. The MPEG–*b*-PCL diblock copolymer is utilized as a carrier for drugs, and for this purpose, it is important to understand the physical–chemical behavior of this moiety at various temperatures and other conditions to design efficient carriers.

## Supporting Information Available

GPC chromatogram for the synthesized MPEG-*b*-PCL diblock copolymer; dynamic light scattering particle size distributions; time evolution of WAXS scattering curves (PDF); SAXS data analysis; and cryo-TEM image at 20 °C (PDF)
