# Supplementary material for: Synthesis and Characterization of a Thermoresponsive Copolymer with an LCST–UCST-like Behavior and Exhibiting Crystallization
Source: ACS Omega. 2023 Aug 17;8(34):31145–54. doi: 10.1021/acsomega.3c03162 (PMC10468772; doi:10.1021/acsomega.3c03162)
Supplement: Supplementary file 1 — ao3c03162_si_001.pdf [file ao3c03162_si_001.pdf]

Supporting information

Synthesis and characterization of a Thermoresponsive Copolymer with an LCST-UCST-Like Behavior and Exhibiting Crystallization

Natalie Solfrid Gjerde, Alessandra Del Giudice, Kaizheng Zhu, Kenneth D. Knudsen*, Luciano Galantini, Karin Schillén, and Bo Nyström*

Natalie Solfrid Gjerde
Department of Chemistry, ‘‘Sapienza’’ University of Rome, P.O. Box 34-Roma 62, Piazzale A. Moro 5, I-00185 Roma, Italy
E-mail: nataliesolfrid.gjerde@uniroma1.it

Alessandra Del Giudice
Department of Chemistry, ‘‘Sapienza’’ University of Rome, P.O. Box 34-Roma 62, Piazzale A. Moro 5, I-00185 Roma, Italy
E-mail: alessandra.delgiudice@uniroma1.it

Kaizheng Zhu
Faculty of Engineering, Østfold University College, P.O. Box 700, 1757, Halden, Norway
E-mail: kaizheng.zhu@kjemi.uio.no

Kenneth D. Knudsen
Institute for Energy Technology, P. O. Box 40, N-2027 Kjeller, Norway
E-mail: kenneth.knudsen@ife.no

Luciano Galantini
Department of Chemistry, ‘‘Sapienza’’ University of Rome, P.O. Box 34-Roma 62, Piazzale A. Moro 5, I-00185 Roma, Italy
E-mail: luciano.galantini@uniroma1.it

Karin Schillén
Division of Physical Chemistry, Department of Chemistry, Lund University, P.O. Box 124, SE-221 00 Lund, Sweden
E-mail: Karin.Schillen@fkem1.lu.se

Bo Nyström
Department of Chemistry, University of Oslo, P.O. Box 1033, Blindern, N-0315 Oslo, Norway
E-mail: bo.nystrom@kjemi.uio.no

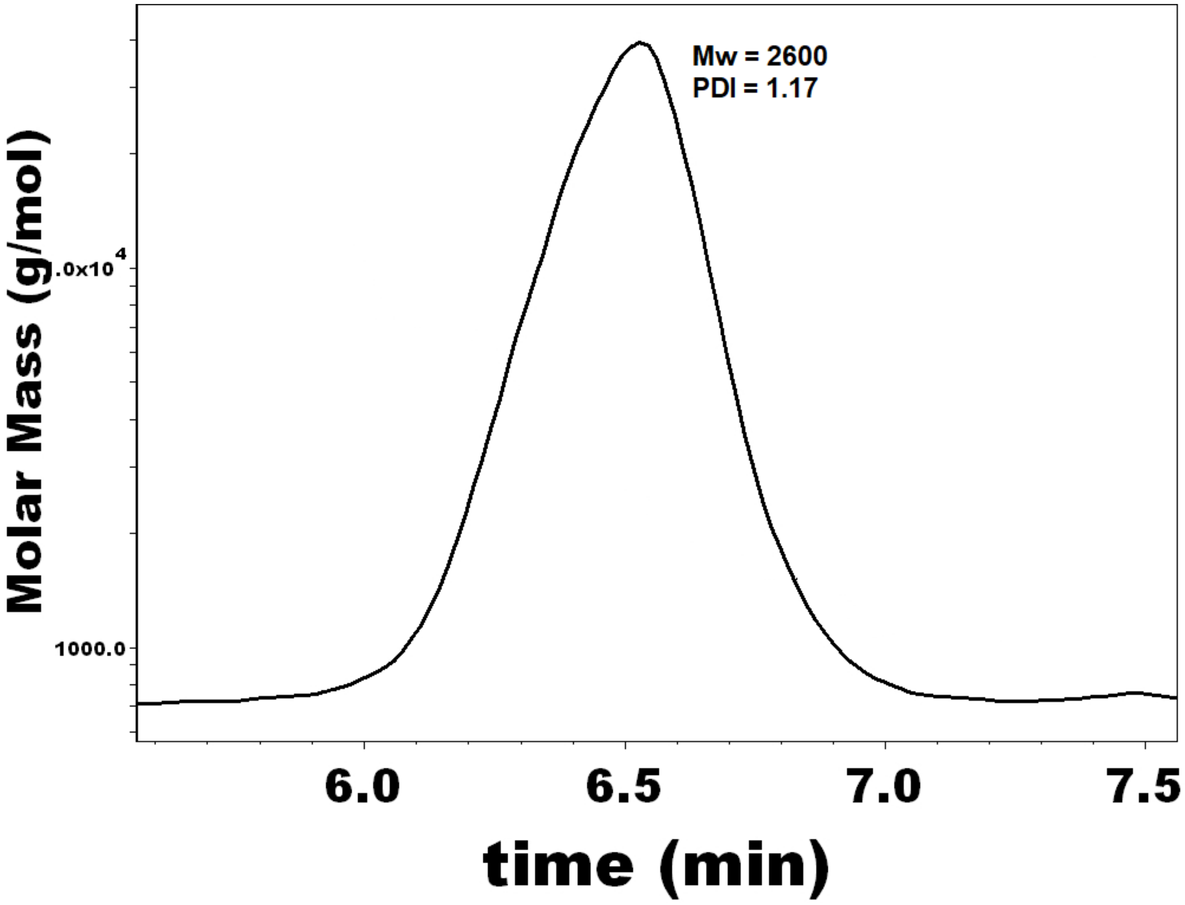

Figure S1. GPC chromatogram for the synthesized MPEG-*b*-PCL diblock copolymer (35 ^{o}C, 10 mg/ml with eluent THF and flow rate of 0.5 ml/min).

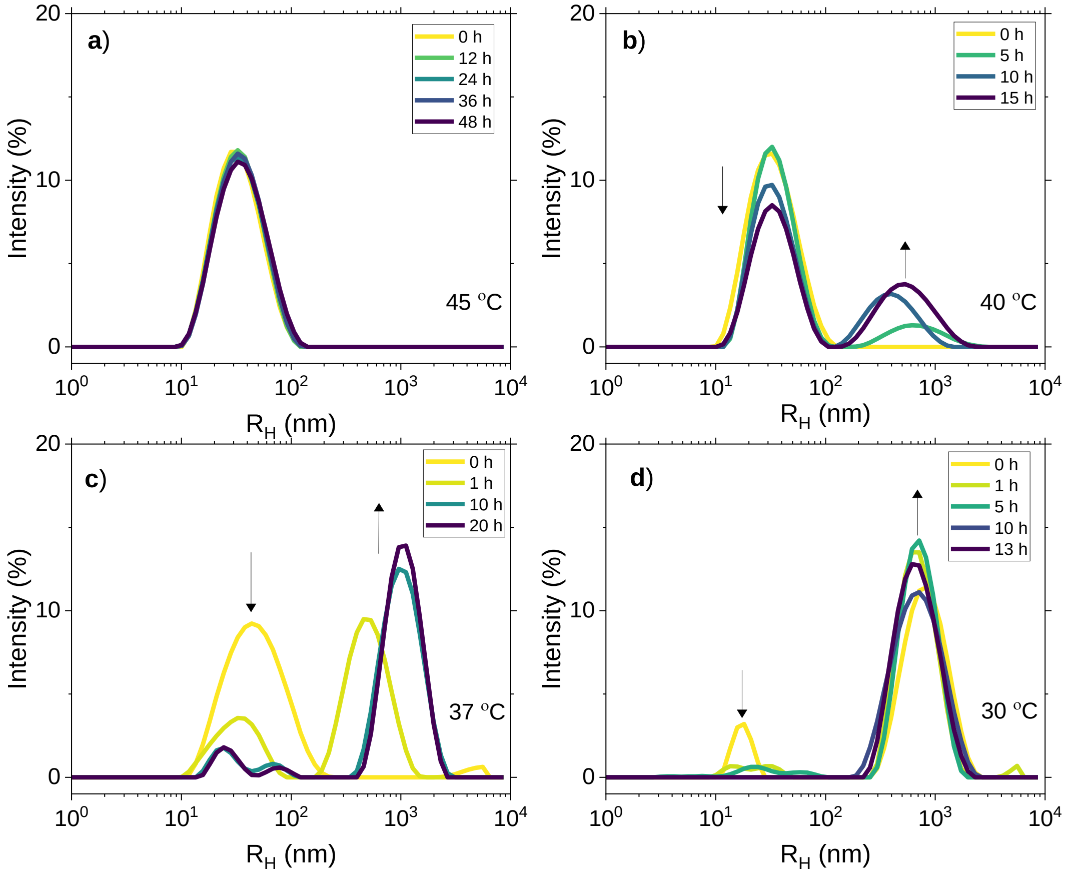

Figure S2. Distributions of the apparent hydrodynamic radii (R_H) from DLS measurements on a 1 wt% MPEG-*b*-PCL aqueous solution at the temperatures and times indicated. The size distribution was calculated with the aid of a non-negative least squares fitting approach. a) 45 °C, b) 40 °C, c) 37 °C, and d) 30 °C.

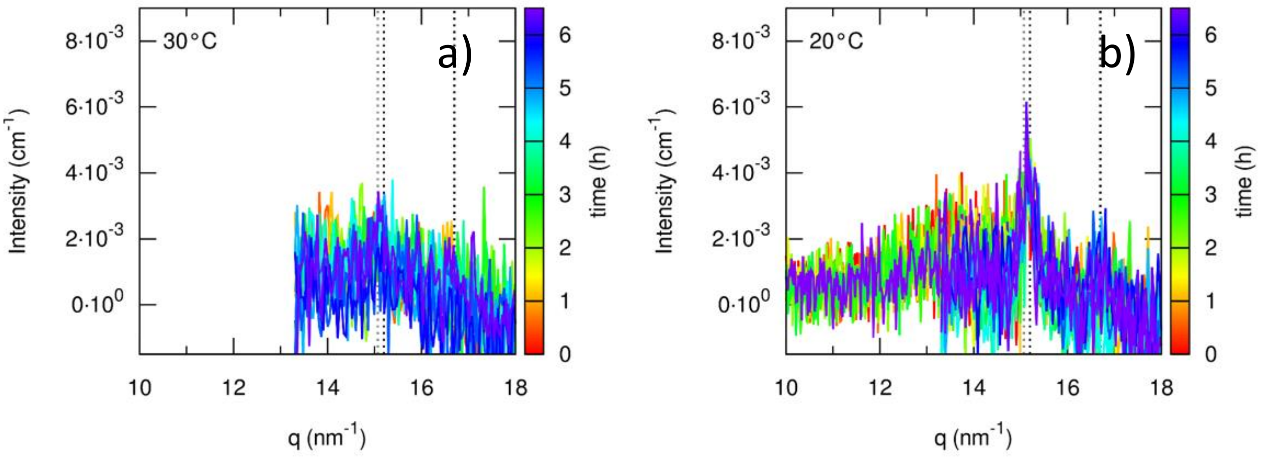

Figure S3. Time evolution of WAXS scattering curves of 1 wt% MPEG-*b*-PCL in water at **a)** 30 °C and **b)** 20 °C.

<u>SAXS data analysis</u>

The SAXS results were analyzed by fitting the data with a spherical core-shell model at 45 °C and a cylindrical core-shell model at 30 °C. The following function was employed to express the form factor for the spherical model within the SASview programming environment:

$$\mathrm{P}(q) = \frac{\mathrm{scale}}{\mathrm{V_t}}\mathrm{F}^2(\mathrm{q}) + \mathrm{background} \qquad \text{(S1)}$$

$$\mathrm{F}^2(\mathrm{q}) = \frac{3}{\mathrm{V_t}}\left[\mathrm{V_c}(\rho_c - \rho_s)\frac{\sin(\mathrm{qr_c}) - \mathrm{qr_c}\cos(\mathrm{qr_c})}{(\mathrm{qr_c})^3} + \mathrm{V_t}(\rho_s - \rho_{solv})\frac{\sin(\mathrm{qr_t}) - \mathrm{qr_t}\cos(\mathrm{qr_t})}{(\mathrm{qr_t})^3}\right] \qquad \text{(S2)}$$

Here V_t is the volume of the whole particle, V_c is the volume of the core, r_t = radius + thickness is the radius of the whole particle, r_c is the radius of the core, ρ_c is the scattering length density of the core, ρ_s is the scattering length density of the shell, and ρ_{solv} is the scattering length density of the solvent.

For the cylindrical model the following function was employed:

$$\mathrm{P}(q) = \frac{\mathrm{scale}}{\mathrm{V_t}}\mathrm{F}^2(\mathrm{q}, \alpha)\sin(\alpha) + \mathrm{background} \qquad \text{(S3)}$$

$$F(q,\alpha) = V_c(\rho_c - \rho_s)\frac{\sin\left(\frac{1}{2}qL\cos(\alpha)\right)\ 2J_1(qr_c\sin(\alpha))}{\frac{1}{2}qL\cos(\alpha)\ qr_c\sin(\alpha)} + V_s(\rho_s - \rho_{solv})\frac{\sin\left(q(\frac{1}{2}L + t)\cos(\alpha)\right)\ 2J_1(q(r_c + t)\sin(\alpha))}{q(\frac{1}{2}L + t)\cos(\alpha)\ q(r_c + t)\sin(\alpha)}$$

(S4)

Here α is the angle between the axis of the cylinder and the scattering vector *q*, *L* is the length of the cylinder, and *t* is the thickness of the shell. J_1 is the first order Bessel function.

The value for the x-ray scattering length density (SLD) of MPEG and PCL were calculated to 11.4 $\cdot 10^{-6}$ Å^{-2} and 10.8 $\cdot 10^{-6}$ Å^{-2}, respectively, and these values were fixed in the modelling. The SLD for the solvent (water) is 9.35 $\cdot 10^{-6}$ Å^{-2}. A structure factor was not included in the modelling (equivalent to setting S(q)=1), due to the low concentration of the samples.

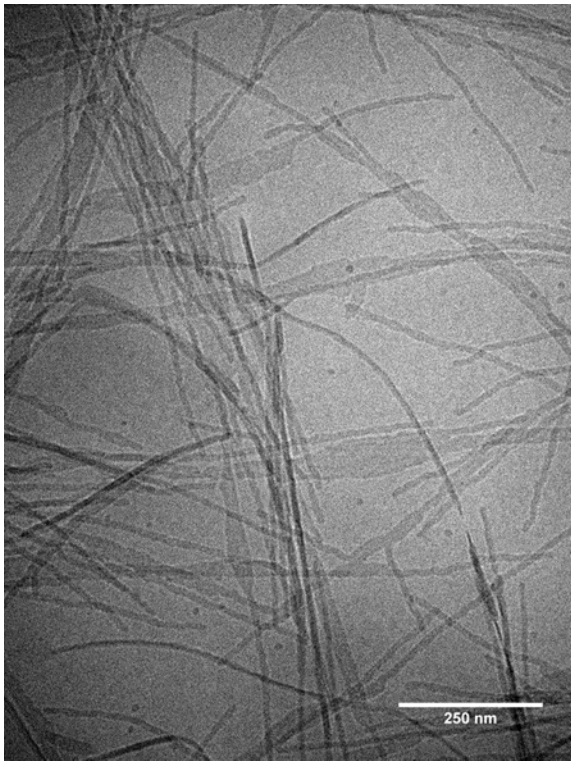

Figure S4. Representative cryo-TEM image of a 1 wt% MPEG-*b*-PCL aqueous solution at 20 ^{o}C.
